# Supplementary material for: ARIH1 signaling promotes anti-tumor immunity by targeting PD-L1 for proteasomal degradation
Source: Nat Commun. 2021 Apr 20;12:2346. doi: 10.1038/s41467-021-22467-8 (PMC8058344; doi:10.1038/s41467-021-22467-8)
Supplement: Supplementary file 1 — Supplementary Information [file 41467_2021_22467_MOESM1_ESM.pdf]

ARIH1 Signaling Promotes Anti-tumor Immunity by Targeting PD-L1 for Proteasomal Degradation

Supplementary information

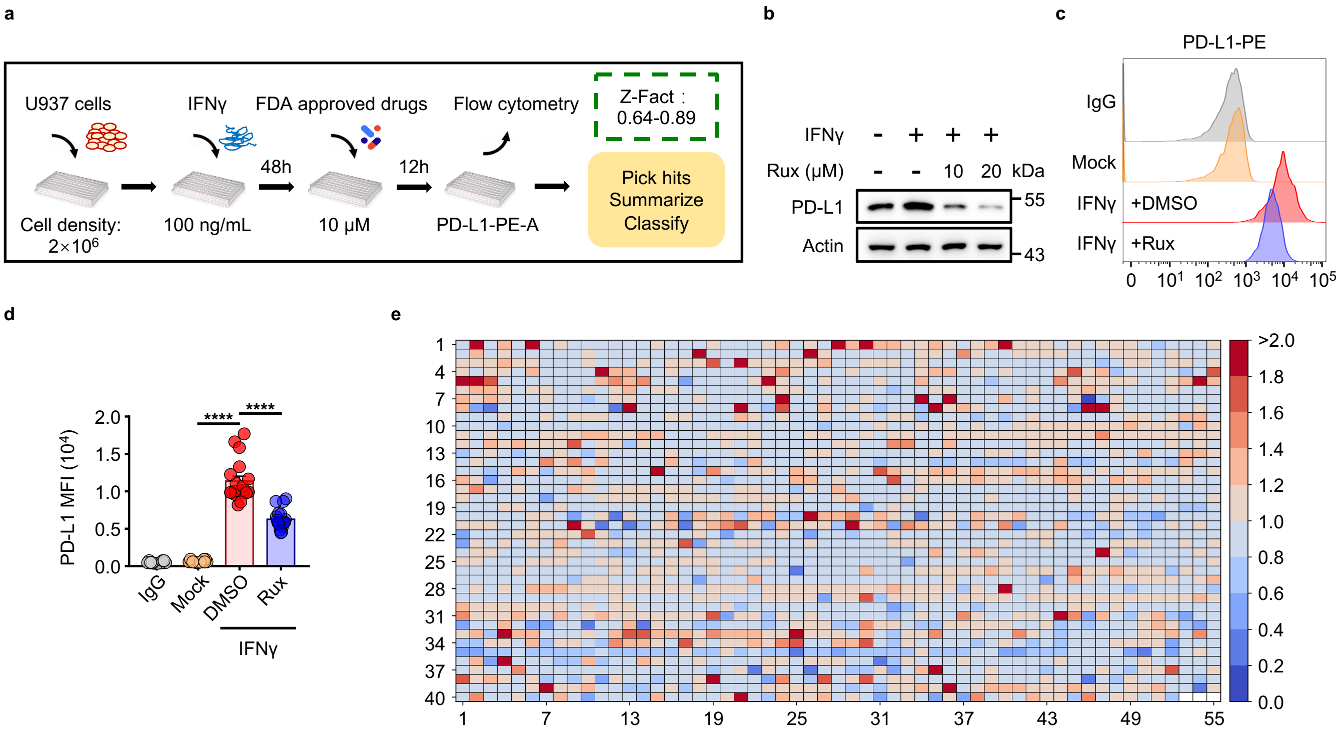

Supplementary Figure 1. *In vitro* drug screen for modulation of membranal PD-L1 levels. **a**, Schematic representation of the drug screening based on membrane PD-L1 detected by flow cytometry. U937 cells were incubated with 100 ng/mL IFNγ for 48 h, then treated with 2125 FDA-approved drugs or drug candidates (10 μM) for 12 h; Ruxolitinib (Rux) was set as a positive control. This image was created by the first author. **b**, Immunoblot of PD-L1 in U937 cells treated with 100 ng/mL IFNγ for 48 h and 10 μM Ruxolitinib for 12 h. **c-d**, MFI (**c**) and relative quantification (**d**) of PD-L1 in U937 cells treated with 100 ng/mL IFNγ for 48 h and 10 μM Ruxolitinib for 12 h. Data represent means ± SEM, n = 18, 6 independent repeats, ****$P < 0.0001$. **e**, A heatmap of membrane PD-L1 treated with each compound from FDA-library compared with baseline controls (DMSO). PD-L1 increase is shown in red shades and a decrease in blue shades. Source data are provided as a Source Data file.

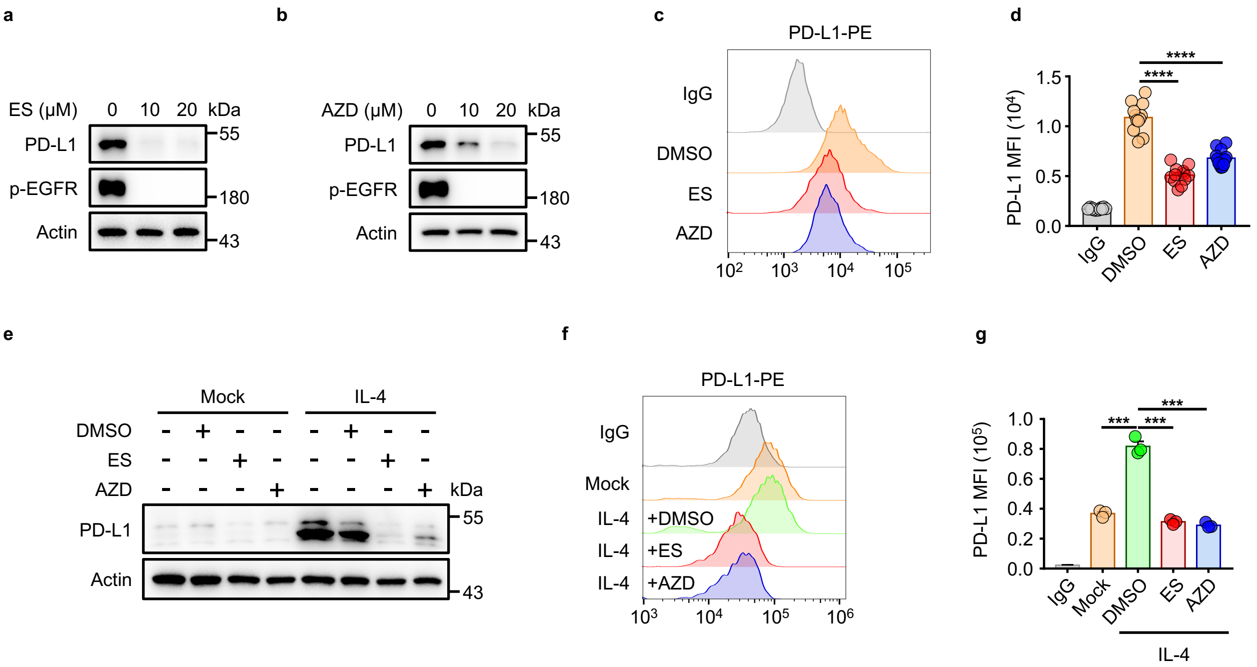

Supplementary Figure 2. ES-072 and AZD9291 modulate membrane PD-L1 levels. a-d, Membrane PD-L1 detection by immunoblot assay (**a** and **b**) and flow cytometry analysis (MFI, **c** and **d**) in H1975 cells treated with 10 μM ES-072 or 10 μM AZD9291 for 24 h. Data represent means ± SEM, n = 12, 4 independent repeats, ****P < 0.0001. **e-g**, Membrane PD-L1 detection by immunoblot assay (**e**) and flow cytometry analysis (MFI, **f** and **g**) in PDMs treated with 100 ng/mL IL-4, 10 μM ES-072 or 10 μM AZD9291 for 48 h. Data represent means ± SEM, n = 3, ***P < 0.001 (P = 0.0002; P = 0.0001; P = 0.0001). Source data are provided as a Source Data file.

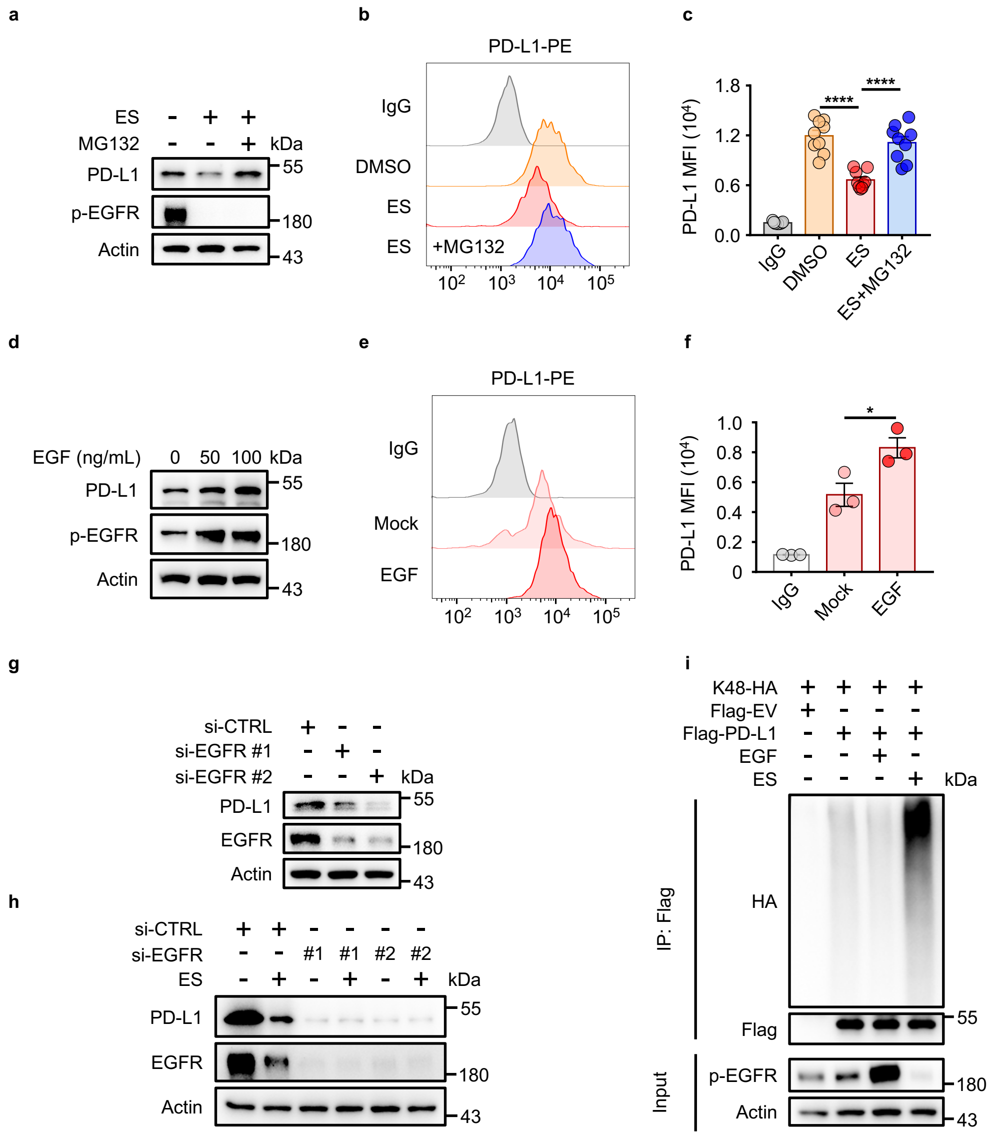

Supplementary Figure 3. Activation of EGFR promotes the stability of membranal PD-L1. **a**, Immunoblot of PD-L1 in H1975 cells treated with 10 μM ES-072 and/or 10 μM MG132 for 6 h. **b-c**, MFI (**b**) and relative quantification (**c**) of PD-L1 in H1975 cells treated with 10 μM ES-072 and/or 10 μM MG132 for 6 h. Data represent means ± SEM, n = 9, 3 independent repeats, ****P < 0.0001. **d-f**, Serum-starved H1975 cells were treated with EGF at indicated concentrations for 48 h. Immunoblots of PD-L1 and p-EGFR (**d**), flow cytometry analysis of MFI (**e**) and relative quantification (**f**) of PD-L1 were performed. Data represent means ± SEM, n = 3, *P < 0.05 (P = 0.037). **g**, Immunoblot of PD-L1 in HEK293T cells transfected with non-targeting siRNA (CTRL) or EGFR-siRNAs. **h**, Immunoblots of PD-L1 and EGFR in H1975 cells transfected with EGFR-siRNAs and treated with or without 10 μM ES-072 for 12 h. **i**, Co-IP analysis for the interaction of K48-ubiquitin (HA) and PD-L1 (Flag) in HEK293T cells transfected with K48-HA and Flag-PD-L1 and treated with EGF (100 ng/mL) or ES-072 (10 μM) for 24 h. Flag-tagged empty vector (Flag-EV) was transfected as a negative control. Source data are provided as a Source Data file.

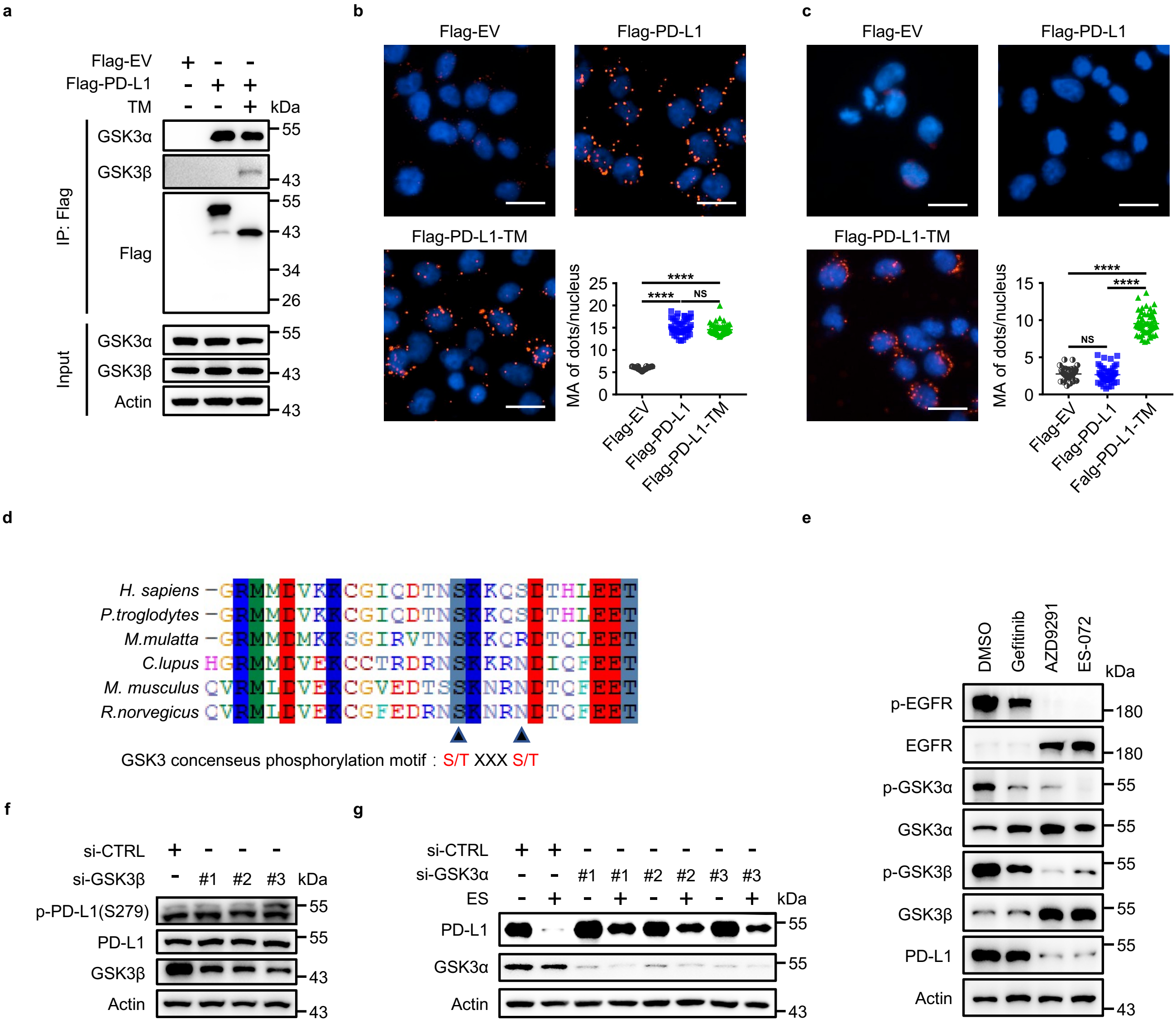

Supplementary Figure 4. GSK3α phosphorylates PD-L1 and decreases PD-L1 stability. a, Co-immunoprecipitation (Co-IP) analysis for the interaction of GSK3α/GSK3β and PD-L1 in HEK293T cells transfected with Flag-tagged-PD-L1 and treated with or without 5 μM tunicamycin (TM, an N-linked glycosylation inhibitor) for 12 h, Flag-tagged empty vector (Flag-EV) was transfected as a negative control. **b-c**, Proximity ligation assay (PLA) analysis for the interaction of GSK3α (**b**) or GSK3β (**c**) and PD-L1 in HEK293T cells treated as **a**. PLA signals are shown in red and the nuclei in blue, scale bar, 20 μm. Quantification for the mean area (MA) of PD-L1/GSK3α (n = 50) or PD-L1/GSK3β (n = 44-50) PLA speckles are indicated by scattergram. Data represent means ± SEM, NS: no significant; *****P* < 0.0001. **d**, Cluster alignment of GSK3 consensus phosphorylation motif on PD-L1. Arrows point to Ser279 and Ser283 of human PD-L1. **e**, Immunoblots of p-EGFR, EGFR, p-GSK3α, GSK3α, p-GSK3β and GSK3β in H1975 cells treated with 10 μM Gefitinib, AZD9291 and ES-072 for 24 h. **f**, Immunoblots of p-PD-L1, PD-L1, and GSK3β in H1975 cells transfected with GSK3β-siRNAs. **g**, Immunoblots of PD-L1 and GSK3α in H1975 cells transfected with GSK3α-siRNAs and treated with or without 10 μM ES-072 for 12 h. Source data are provided as a Source Data file.

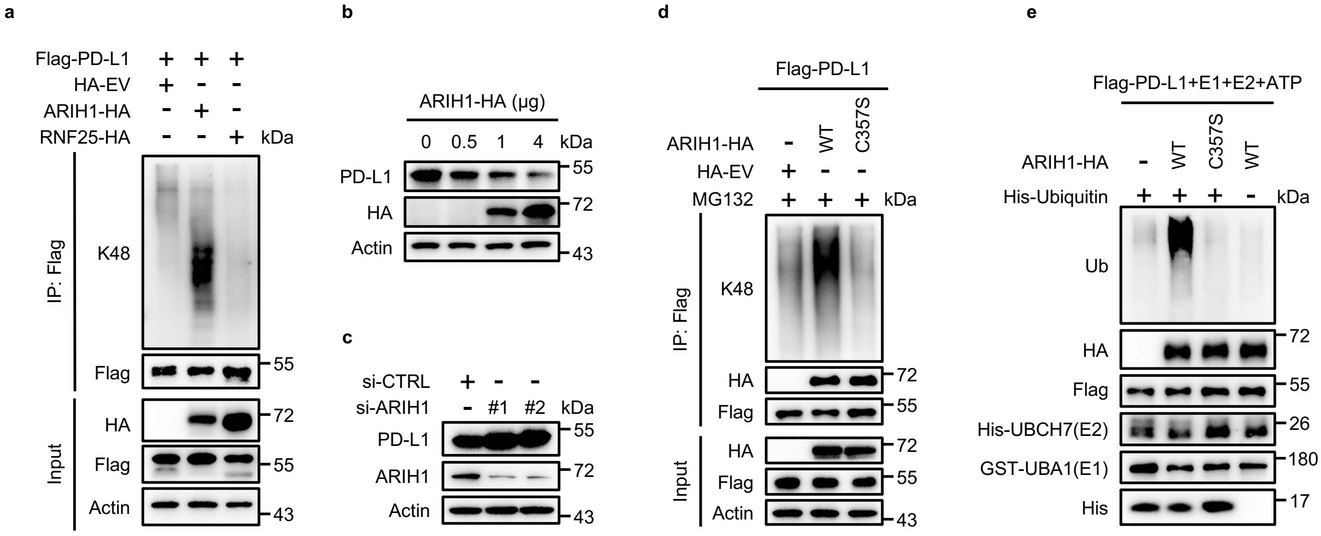

Supplementary Figure 5. ARIH1 mediates PD-L1 ubiquitination and degradation. a, Co-IP analysis for the interaction of K48-ubiquitin and PD-L1 (Flag) in HEK293T cells transfected with Flag-PD-L1 and ARIH1-HA or RNF25-HA. HA-tagged empty vector (HA-EV) was transfected as a negative control. **b-c**, Immunoblots of PD-L1 and ARIH1 (HA) in HEK293T cells transfected with ARIH1-HA (**b**) or ARIH1-siRNAs (**c**). **d**, Co-IP analysis for the interaction of K48-ubiquitin, ARIH1 (HA) and PD-L1 in HEK293T cells transfected with Flag-PD-L1 and ARIH1-HA (WT or C357S), treated with 10 μM MG132 for 6 h. **e**, Recombinant Flag-tagged PD-L1 and HA-tagged ARIH1(WT) or C357S-mutated ARIH1 was purified in HEK293T cells, respectively. An *in vitro* ubiquitination assay was performed either with ubiquitin, UBA1 (E1), UBCH7 (E2), and ARIH1 (E3) or its inactive mutant (C357S) or in the absence of ARIH1 or ubiquitin. Source data are provided as a Source Data file.

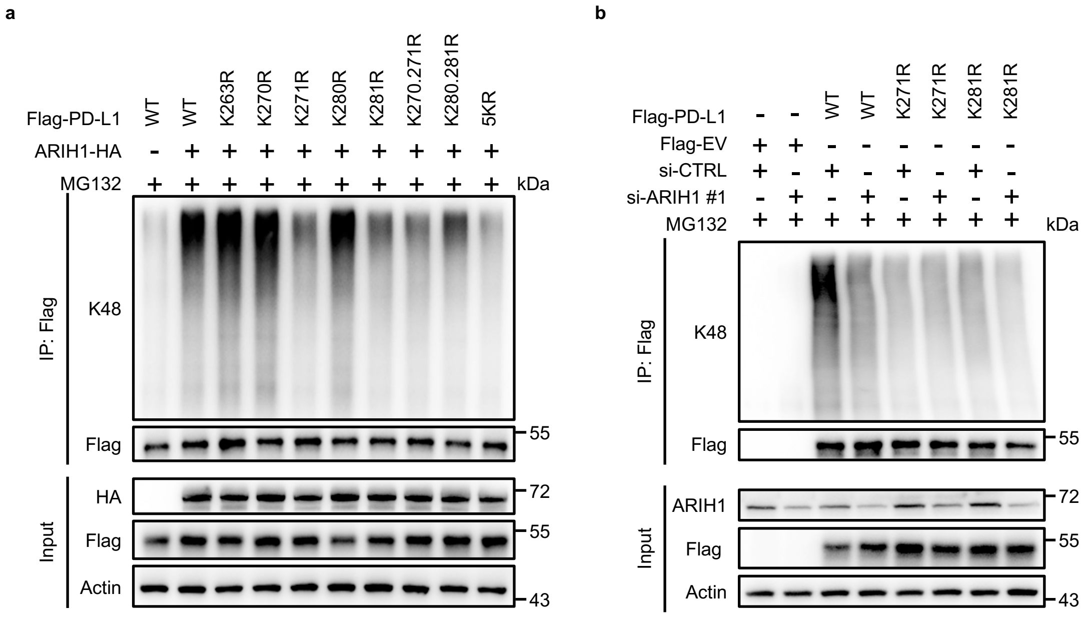

Supplementary Figure 6. K271 and K281 of PD-L1 are responsible for the ubiquitination and degradation mediated by ARIH1. a, Co-IP analysis for the interaction of K48-ubiquitin and PD-L1 in HEK293T cells transfected with Flag-tagged PD-L1 (WT or mutants) and HA-tagged ARIH1 in the presence of MG132 (10 μM) for 6 h. **b**, Co-IP analysis for the interaction of K48-ubiquitin and PD-L1 in HEK293T cells transfected with Flag-tagged PD-L1 (WT or mutants) in the presence or absence of ARIH1-siRNA#1, treated with 10 μM MG132 for 6 h. Flag-tagged empty vector (Flag-EV) and non-targeting siRNA (si-CTRL) were transfected as a negative control. Source data are provided as a Source Data file.

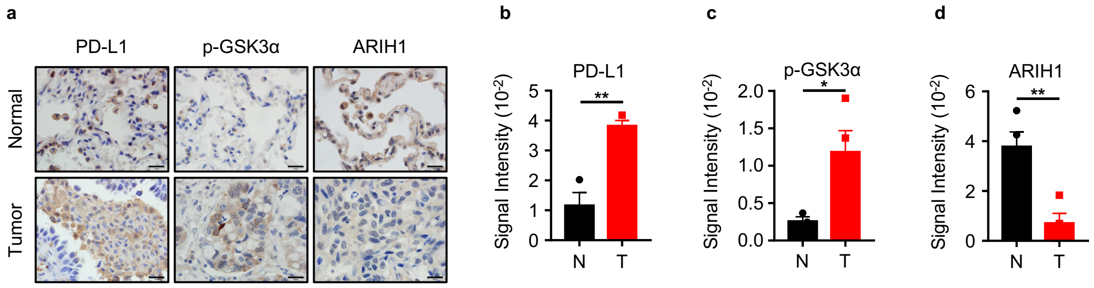

Supplementary Figure 7. The protein levels of PD-L1, p-GSK3α, and ARIH1 in human lung adenocarcinoma biopsies. a, Representative images of PD-L1, p-GSK3α and ARIH1 IHC staining from human alveolar adenocarcinoma and paracancerous normal tissue specimens. Scale bars represent 20 μm. **b-d**, Quantification of IHC analysis for PD-L1 (**b**), p-GSK3α (**c**), and ARIH1 (**d**) (n = 3-4)**.** Data represent means ± SEM. **b**, ***P* < 0.01 (*P* = 0.0048); **c**, **P* < 0.05 (*P* = 0.0415); **d**, ***P* < 0.01 (*P* = 0.0049). Source data are provided as a Source Data file.

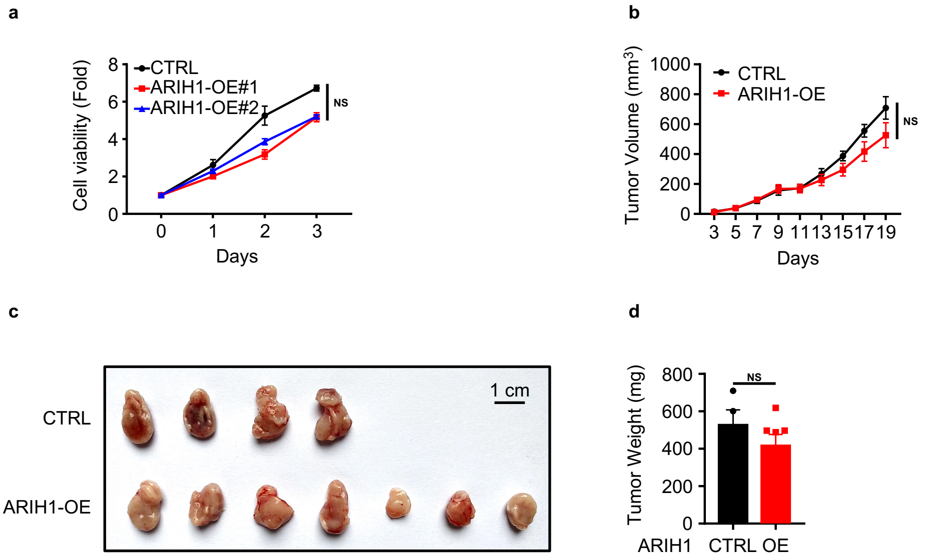

Supplementary Figure 8. Overexpression of ARIH1 showed no effect on the proliferation of 4T1 cells *in vitro* and in immuno-compromised mice. a, 4T1 cells were infected with an empty vector (CTRL) or two different ARIH1 overexpressing lentiviral preparations (OE#1 and OE#2). Cell viability was monitored at indicated time points by an ATP assay. Data represent means ± SEM, n = 8, NS: not significant. **b-d**, Tumor growth (**b-c**) of CTRL (n = 4) and ARIH1-OE cells (n = 7) in nude mice and final tumor weights (**d**). Data represent means ± SEM, NS: not significant. Source data are provided as a Source Data file.

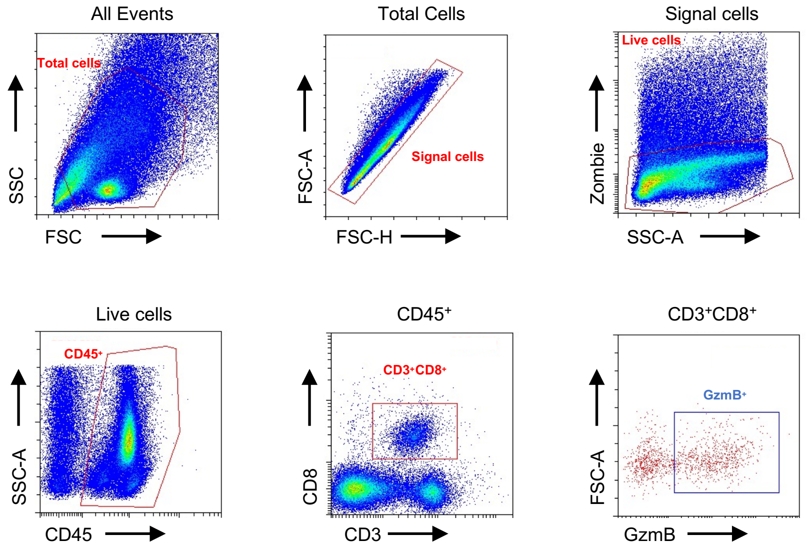

Supplementary Figure 9. Gating procedure for CD8⁺ T cells and GzmB⁺ T cells in tumor microenvironment.

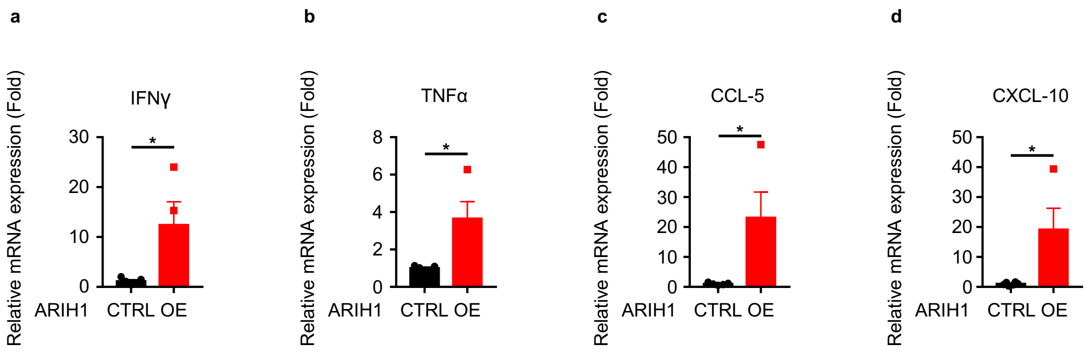

Supplementary Figure 10. qRT-PCR analysis for cytokine mRNA levels in bulk tumor xenografts. a-d, qRT-PCR analysis for the gene expression of IFNγ (**a**), TNFα (**b**), CCL-5 (**c**), and CXCL-10 (**d**) (n = 4-6). Data represent means ± SEM. **a**, **P* < 0.05 (*P* = 0.0283); **b**, **P* < 0.05 (*P* = 0.0126); **c**, **P* < 0.05 (*P* = 0.0221); **d**, **P* < 0.05 (*P* = 0.0122). Source data are provided as a Source Data file.

Catalog Number	Item Name	M.w.	Target	Pathway
S2796	WP1066	356.22	JAK	JAK/STAT
S8057	Pacritinib (SB1518)	472.58	JAK	JAK/STAT
S7024	Stattic	211.19	STAT	JAK/STAT
S2902	S-Ruxolitinib (INCB018424)	306.37	JAK	JAK/STAT
S2789	Tofacitinib (CP-690550,Tasocitinib)	312.37	JAK	JAK/STAT
S7036	XL019	444.53	JAK	JAK/STAT
S2806	CEP-33779	462.57	JAK	JAK/STAT
S7337	SH-4-54	610.59	STAT	JAK/STAT
S7634	Cerdulatinib (PRT062070, PRT2070)	482	JAK	JAK/STAT
S5001	Tofacitinib (CP-690550) Citrate	504.49	JAK	JAK/STAT
S7041	CX-6258 HCl	498.4	Pim	JAK/STAT
S7605	Filgotinib (GLPG0634)	425.5	JAK	JAK/STAT
S2214	AZ 960	354.36	JAK	JAK/STAT
S2692	TG101209	509.67	Flt, JAK, c-RET	JAK/STAT
S2686	NVP-BSK805 2HCl	563.47	JAK	JAK/STAT
S2736	TG101348 (SAR302503)	524.68	JAK	JAK/STAT
S2219	CYT387	414.46	JAK	JAK/STAT
S2670	A-674563	358.44	Akt, CDK, PKA	PI3K/Akt/mTOR
S8019	AZD5363	428.92	Akt	PI3K/Akt/mTOR
S2749	BGT226 (NVP-BGT226)	650.6	PI3K, mTOR	PI3K/Akt/mTOR
S1523	SAR245409 (XL765)	599.66	PI3K, mTOR	PI3K/Akt/mTOR
S2218	PP242	308.34	mTOR	PI3K/Akt/mTOR
S7693	AZD6738	412.51	ATM/ATR	PI3K/Akt/mTOR
S2696	GDC-0980 (RG7422)	498.6	mTOR, PI3K	PI3K/Akt/mTOR
S7675	PF-4989216	380.4	PI3K	PI3K/Akt/mTOR

S7356	HS-173	422.46	PI3K	PI3K/Akt/mTOR
S2227	PIK-294	489.53	PI3K	PI3K/Akt/mTOR
S7317	WZ4003	496.99	AMPK	PI3K/Akt/mTOR
S7798	GNE-317	414.48	PI3K	PI3K/Akt/mTOR
S7016	VS-5584 (SB2343)	354.41	PI3K	PI3K/Akt/mTOR
S2658	GSK2126458 (GSK458)	505.5	PI3K, mTOR	PI3K/Akt/mTOR
S2870	TG100713	254.25	PI3K	PI3K/Akt/mTOR
S2743	PF-04691502	425.48	mTOR, PI3K, Akt	PI3K/Akt/mTOR
S2226	CAL-101 (Idelalisib, GS-1101)	415.42	PI3K	PI3K/Akt/mTOR
S2688	R547	441.45	CDK	Cell Cycle
S2742	PHA-767491	213.24	CDK	Cell Cycle
S1529	Hesperadin	516.65	Aurora Kinase	Cell Cycle
S2768	Dinaciclib (SCH727965)	396.49	CDK	Cell Cycle
S8058	P276-00	438.3	CDK	Cell Cycle
S2679	Flavopiridol HCl	438.3	CDK	Cell Cycle
S2621	AZD5438	371.46	CDK	Cell Cycle
S1524	AT7519	382.24	CDK	Cell Cycle
S7461	LDC000067	370.43	CDK	Cell Cycle
S2683	CHIR-124	419.91	Chk	Cell Cycle
S2718	TAK-901	504.64	Aurora Kinase	Cell Cycle
S7440	LEE011	434.54	CDK	Cell Cycle
S1519	CCT129202	497.02	Aurora Kinase	Cell Cycle
S7158	LY2835219	602.7	CDK	Cell Cycle
S7057	LY2874455	444.31	FGFR	Protein Tyrosine Kinase
S7846	TP-0903	516.06	TAM Receptor	Protein Tyrosine Kinase
S7998	Entrectinib (RXDX-101)	560.64	Trk receptor	Protein Tyrosine Kinase

S2730	Crenolanib (CP-868596)	443.54	PDGFR	Protein Tyrosine Kinase
S7106	AZD3463	448.95	ALK	Protein Tyrosine Kinase
S7358	Poziotinib (HM781-36B)	491.34	EGFR	Protein Tyrosine Kinase
S7083	LDK378	558.14	ALK	Protein Tyrosine Kinase
S7297	AZD9291	499.61	EGFR	Protein Tyrosine Kinase
S2216	Mubritinib (TAK 165)	468.47	HER2	Protein Tyrosine Kinase
ES-0001	ES-072	528.54	EGFR	Protein Tyrosine Kinase
S2201	BMS-794833	468.84	c-Met, VEGFR	Protein Tyrosine Kinase
S7357	PF-562271 HCl	543.95	FAK	Angiogenesis
S7644	PF-431396	506.5	FAK	Angiogenesis
S2672	PF-00562271	665.66	FAK	Angiogenesis
S7545	G-749	521.41	FLT3	Angiogenesis
S7654	Defactinib (VS-6063, PF-04554878)	510.49	FAK	Angiogenesis
S2890	PF-562271	507.49	FAK	Angiogenesis
S2634	DCC-2036 (Rebastinib)	553.59	Bcr-Abl	Angiogenesis
S1537	DMXAA (Vadimezan)	282.29	VDA	Angiogenesis
S7028	IPI-145 (INK1197)	416.86	PI3K	Angiogenesis
S7194	GZD824	724.77	Bcr-Abl	Angiogenesis
S1528	LY2811376	320.36	5-alpha Reductase	Proteases
S1538	Telaprevir (VX-950)	679.85	HCV Protease	Proteases
S1290	Celastrol	450.61	Others	Proteases
S7386	MG-101 (ALLN)	383.53	Cysteine Protease	Proteases

S7172	ONX-0914 (PR-957)	580.67	Proteasome	Proteases
S7424	PD 151746	237.25	Cysteine Protease	Proteases
S7462	PI-1840	394.47	Proteasome	Proteases
S7379	E-64	357.41	Cysteine Protease	Proteases
S4282	Nelfinavir Mesylate	663.89	HIV Protease	Proteases
S7396	Calpeptin	362.46	Cysteine Protease	Proteases
S7409	Anisomycin	265.3	JNK	MAPK
S7334	ERK5-IN-1	638.81	ERK	MAPK
S2134	AZD8330	461.23	MEK	MAPK
S7108	LGX818	540.01	Raf	MAPK
S8015	CEP-32496	517.46	Raf	MAPK
S2807	Dabrafenib (GSK2118436)	519.56	Raf	MAPK
S7842	LY3009120	424.51	Raf	MAPK
S8041	Cobimetinib (GDC-0973, RG7420)	531.31	MEK	MAPK
S2161	RAF265 (CHIR-265)	518.41	Raf, VEGFR	MAPK
S2673	Trametinib (GSK1120212)	615.39	MEK	MAPK
S7094	PF-3758309	490.62	PAK	Cytoskeletal Signaling
S7122	XL888	503.64	HSP (e.g. HSP90)	Cytoskeletal Signaling
S7097	HSP990 (NVP-HSP990)	379.39	HSP (e.g. HSP90)	Cytoskeletal Signaling
S3020	Romidepsin (FK228, Depsipeptide)	540.7	HDAC	Cytoskeletal Signaling
S7340	CH5138303	415.9	HSP (e.g. HSP90)	Cytoskeletal Signaling
S7336	CW069	500.33	Microtubule Associated	Cytoskeletal Signaling
S7751	VER155008	556.4	HSP (e.g. HSP90)	Cytoskeletal Signaling
S7355	ARQ 621	552.43	Kinesin	Cytoskeletal Signaling
S7494	INH6	322.42	Microtubule Associated	Cytoskeletal Signaling
S7458	VER-49009	387.82	HSP	Cytoskeletal Signaling

S2851	Baricitinib (LY3009104, INCB028050)	371.42	JAK	Epigenetics
S7360	OTX015	491.99	BET	Epigenetics
S7620	GSK1324726A (I-BET726)	434.91	Epigenetic Reader Domain	Epigenetics
S7304	CPI-203	399.9	Epigenetic Reader Domain	Epigenetics
S7438	ME0328	321.37	PARP	Epigenetics
S7110	(+)-JQ1	456.99	BET	Epigenetics
S7476	MG149	340.46	Histone Acetyltransferase	Epigenetics
S7189	I-BET-762	423.9	Epigenetic Reader Domain	Epigenetics
S7315	PFI-3	321.37	Epigenetic Reader Domain	Epigenetics
S7136	CGK 733	555.84	ATM/ATR	DNA Damage
S7757	6-Thio-dG	283.31	DNA/RNA Synthesis	DNA Damage
S7718	BMH-21	360.41	DNA/RNA Synthesis	DNA Damage
S2423	(S)-10-Hydroxycamptothecin	364.35	Topoisomerase	DNA Damage
S7449	CRT0044876	206.15	DNA/RNA Synthesis	DNA Damage
S7029	AZD2461	395.43	PARP	DNA Damage
S1714	Gemcitabine	263.2	Others	DNA Damage
S7445	E3330	378.46	DNA/RNA Synthesis	DNA Damage
S8065	Nutlin-3b	581.49	Mdm2	Apoptosis
S2812	AT101	578.61	Bcl-2	Apoptosis
S7489	YH239-EE	504.41	Mdm2	Apoptosis
S7597	BV-6	1205.57	IAP	Apoptosis
S8000	Tenovin-1	369.48	p53	Apoptosis
S7326	Tasisulam	415.11	Caspase	Apoptosis
S7912	PD-1/PD-L1 inhibitor 2	419.52	Others	Apoptosis
S2781	RITA (NSC 652287)	292.37	p53	Apoptosis
S4075	Zinc Pyrithione	317.7	Proton Pump	Transmembrane Transporters
S7046	Brefeldin A	280.36	ATPase	Transmembrane Transporters

S7266	Golgicide A	284.3	ATPase	Transmembrane Transporters
S2233	Esomeprazole Sodium	367.4	ATPase	Transmembrane Transporters
S1979	Amiodarone HCl	681.77	Potassium Channel	Transmembrane Transporters
S1293	Cilnidipine	492.52	Calcium Channel	Transmembrane Transporters
S7707	Verdinexor (KPT-335)	442.32	CRM1	Transmembrane Transporters
S8021	Vortioxetine (Lu AA21004) HBr	379.36	5-HT Receptor	Neuronal Signaling
S2722	JTC-801	447.96	Opioid Receptor	Neuronal Signaling
S3061	Epinephrine HCl	219.67	Adrenergic Receptor	Neuronal Signaling
S7366	LY2119620	437.94	AChR	Neuronal Signaling
S1071	HA14-1	409.23	Bcl-2	Neuronal Signaling
S3005	Paroxetine HCl	365.83	5-HT Receptor	Neuronal Signaling
S7442	WS6	568.59	IκB/IKK	NF-κB
S7441	WS3	569.58	IκB/IKK	NF-κB
S2882	IKK-16 (IKK Inhibitor VII)	483.63	IKK	NF-κB
S2824	TPCA-1	279.29	IKK	NF-κB
S2864	IMD 0354	383.67	IKK	NF-κB
S5002	Fingolimod (FTY720) HCl	343.9	S1P Receptor, Bcr-Abl, PKC	GPCR & G Protein
S7138	BMS-833923	473.57	Hedgehog/Smoothened	GPCR & G Protein
S7588	Reversine	393.23	Adenosine Receptor	GPCR & G Protein
S4296	Salmeterol Xinafoate	603.75	Adrenergic Receptor	GPCR & G Protein
S7465	FTI 277 HCl	484.07	Transferase	Metabolism
S7467	LB42708	555.46	Transferase	Metabolism
S2187	Avasimibe	501.72	P450	Metabolism
S7699	Liproxstatin-1	340.85	Ferroptosis	Metabolism
S7142	NSC697923	267.26	E2 conjugating	Ubiquitin
S4920	b-AP15	419.39	DUB	Ubiquitin

S7130	PR-619	223.28	DUB	Ubiquitin
S7399	FLI-06	438.52	Gamma-secretase	Stem Cells & Wnt
S7490	WIKI4	521.59	Wnt/beta-catenin	Stem Cells & Wnt
S7147	LDN-212854	406.48	TGF-beta/Smad	TGF-beta/Smad
S7119	Go6976	377.42	PKC	TGF-beta/Smad
S7367	GNE-0877	339.32	Others	Autophagy
S7368	GNE-9605	449.83	LRRK2	Autophagy
S2149	GSK1292263	456.56	GPR	Endocrinology & Hormones

Supplementary Table 1. 160 FDA approved drugs or drug candidates that reduce membrane PD-L1 level.

GSK3α		
**Compound ID**	**Operator**	**IC50 (nM)**
ES-072	>	40000
LY2090314	=	0.98
ES-072	>	40000
LY2090314	=	0.81
ES-072	>	40000
LY2090314	=	0.84
Staurosporine	=	46
Staurosporine	=	46

Supplementary Table 2. *In vitro* kinase specificity profiling of ES-072 and LY2090314 for GSK3α.

Compound		CDK4	EGFR	EGFR L858R	EGFR T790M
ES-072	Bottom		2.2	0.1	
	Top		98	99	
	IC50 (nM)	>10000	8.9	2.7	<0.5
	Hill		1.1	1.7	
Staurosporine	Bottom	-5.4	2.2	-1.0	1.3
	Top	103	102	97	100
	IC50 (nM)	25	41	12	0.30
	Hill	0.9	0.8	1.1	1.0
**Compound**		**KDR(VEGFR2)**	**CMET**	**CDK6**	**PDGFRa**
ES-072	Bottom	6.1			15
	Top	100			100
	IC50 (nM)	5234	>10000	>10000	3847
	Hill	1.2			1.3
Staurosporine	Bottom	4.5	-7.4	0.4	1.1
	Top	101	100	103	99
	IC50 (nM)	5.2	107	254	0.45
	Hill	1.0	1.0	0.8	1.6
**Compound**		**JAK2**	**IGF1R**	**HER2**	**L858R/T790M**
ES-072	Bottom	9.5	6.6	2.9	
	Top	100	98	101	
	IC50 (nM)	4964	127	147	1.75
	Hill	0.86	1.0	1.3	
Staurosporine	Bottom	1.1	8.2	4.2	
	Top	100	97	100	
	IC50 (nM)	0.30	88	415	
	Hill	1.1	1.1	0.73	

Supplementary Table 3. *In vitro* kinase specificity profiling for ES-072.

Positions	ES	NC	ES/NC
S79	88301282.05	68965843.02	1.28
S93	301602564.10	197790697.67	1.52
T148	0.00	0.00	-
S149	0.00	0.00	-
S169	5003205.13	0.00	-
S170	0.00	2900145.35	0.00
S176	0.00	2260537.79	0.00
S195	0.00	0.00	-
S196	0.00	0.00	-
S279	13245512.82	2648909.88	5.00
S283	1576298076.92	763372093.02	2.06
T290	12242307.69	6262936.05	1.95

Supplementary Table 4. The PD-L1 phosphorylation sites discovered by MS-MS analysis.

Genes	siRNA Sequences
β-TrCP#1	5’-GGAAGAUAAUACCAGAGAAGA-3’
β-TrCP#2	5’-GCACUUGCGUUUCAAUAAUUU-3’
EGFR#1	5’-CACAGUGGAGCGAAUUCCUUU-3’
EGFR#2	5’-GAGGAAAUAUGUACUACGAAA-3’
GSK3α#1	5’-CCUGGACAAAGGUGUUCAAAU-3’
GSK3α#2	5’-CCAACUACACGGAGUUCAAGU-3’
GSK3α#3	5’-GGGUGUAAAUAGAUUGUUAUA-3’
ARIH1#1	5’-CGAGAUAUUUCCCAAGAUUUU-3’
ARIH1#2	5’-CCAUGUUGUUAAAGUCCAAUA-3’
control	5’-UUCUCCGAACGUGUCACGUTT-3’

Supplementary Table 5. The siRNA sequences used in this study.

Genes	Primers
mActb-F	5’-GGCTGTATTCCCCTCCATCG-3’
mActb-R	5’-CCAGTTGGTAACAATGCCATGT-3’
mIfng-F	5’-ACAGCAAGGCGAAAAAGGATG-3’
mIfng-R	5’-TGGTGGACCACTCGGATGA-3’
mTnfa-F	5’-CCCTCACACTCAGATCATCTTCT-3’
mTnfa-R	5’-GCTACGACGTGGGCTACAG-3’
mCcl5-F	5’-GCTGCTTTGCCTACCTCTCC-3’
mCcl5-R	5’-TCGAGTGACAAACACGACTGC-3’
mCxcl10-F	5’-TGAATCCGGAATCTAAGACCATCAA-3’
mCxcl10-R	5’-AGGACTAGCCATCCACTGGGTAAAG-3’

Supplementary Table 6. The qRT-PCR primers used in this study.
