# Supplementary material for: ARIH1 signaling promotes anti-tumor immunity by targeting PD-L1 for proteasomal degradation
Source: Nat Commun. 2021 Apr 20;12:2346. doi: 10.1038/s41467-021-22467-8 (PMC8058344; doi:10.1038/s41467-021-22467-8)
Supplement: Supplementary file 3 — Reporting Summary [file 41467_2021_22467_MOESM3_ESM.pdf]

nature research

Corresponding author(s): Hongguang Xia

Last updated by author(s): Feb 18, 2021

Reporting Summary

Nature Research wishes to improve the reproducibility of the work that we publish. This form provides structure for consistency and transparency in reporting. For further information on Nature Research policies, see our Editorial Policies and the Editorial Policy Checklist.

Statistics

For all statistical analyses, confirm that the following items are present in the figure legend, table legend, main text, or Methods section.

n/a	Confirmed	
☐	☒	The exact sample size (n) for each experimental group/condition, given as a discrete number and unit of measurement
☐	☒	A statement on whether measurements were taken from distinct samples or whether the same sample was measured repeatedly
☐	☒	The statistical test(s) used AND whether they are one- or two-sided<br>*Only common tests should be described solely by name; describe more complex techniques in the Methods section.*
☒	☐	A description of all covariates tested
☒	☐	A description of any assumptions or corrections, such as tests of normality and adjustment for multiple comparisons
☐	☒	A full description of the statistical parameters including central tendency (e.g. means) or other basic estimates (e.g. regression coefficient) AND variation (e.g. standard deviation) or associated estimates of uncertainty (e.g. confidence intervals)
☐	☒	For null hypothesis testing, the test statistic (e.g. F, t, r) with confidence intervals, effect sizes, degrees of freedom and P value noted<br>*Give P values as exact values whenever suitable.*
☒	☐	For Bayesian analysis, information on the choice of priors and Markov chain Monte Carlo settings
☒	☐	For hierarchical and complex designs, identification of the appropriate level for tests and full reporting of outcomes
☒	☐	Estimates of effect sizes (e.g. Cohen's d, Pearson's r), indicating how they were calculated

Our web collection on statistics for biologists contains articles on many of the points above.

Software and code

Policy information about availability of computer code

Data collection	Flow cytometry data were collected using CytExpert v2.3 (Beckman Coulter).<br>Imaging data were collected using cytation 3.
Data analysis	GraphPad Prism 7.0 was used for statistical analyses<br>Gen5 2.0 was used for cytation 3 images processing<br>CytExpert v2.3 and FlowJo X were used for the analysis and quantification of flow cytometry

For manuscripts utilizing custom algorithms or software that are central to the research but not yet described in published literature, software must be made available to editors and reviewers. We strongly encourage code deposition in a community repository (e.g. GitHub). See the Nature Research guidelines for submitting code & software for further information.

Data

Policy information about availability of data

All manuscripts must include a data availability statement. This statement should provide the following information, where applicable:

- Accession codes, unique identifiers, or web links for publicly available datasets
- A list of figures that have associated raw data
- A description of any restrictions on data availability

DATA AVAILABILITY STATEMENT
The data that support the findings of this study are available from the corresponding author upon reasonable request. Source data are provided with the paper.

Field-specific reporting

Please select the one below that is the best fit for your research. If you are not sure, read the appropriate sections before making your selection.

☒ Life sciences ☐ Behavioural & social sciences ☐ Ecological, evolutionary & environmental sciences

For a reference copy of the document with all sections, see nature.com/documents/nr-reporting-summary-flat.pdf

Life sciences study design

All studies must disclose on these points even when the disclosure is negative.

Sample size	Sample size estimates has been performed on previous experience to obtain statistical significance and reproducibility. For in vitro experiments such as Western blot, qPCR, IHC, flow cytometry and PLA assay, at least three samples were used per group for minimal statistics requirements. For in vivo studies, the sample size was determined to be sufficient to obtain the statistical difference between groups.
Data exclusions	There are no data exclusions
Replication	All experiments underlying main conclusions of this study have been successfully replicated multiple times and corroborated by several models. All the western blot, qPCR, IHC, flow cytometry and PLA assay were carried out at least three independent times with the same results.
Randomization	Samples and organisms were randomly allocated to experimental groups. No specific randomization protocol has been used. Mice were age- and sex matched.
Blinding	No specific blinding was applied since all experiments were assigned into groups including relevalant controls and analysis was done objectively and without bias.

Reporting for specific materials, systems and methods

We require information from authors about some types of materials, experimental systems and methods used in many studies. Here, indicate whether each material, system or method listed is relevant to your study. If you are not sure if a list item applies to your research, read the appropriate section before selecting a response.

Materials & experimental systems

n/a	Involved in the study	
☐	☒	Antibodies
☐	☒	Eukaryotic cell lines
☒	☐	Palaeontology and archaeology
☐	☒	Animals and other organisms
☐	☒	Human research participants
☒	☐	Clinical data
☒	☐	Dual use research of concern

Methods

n/a	Involved in the study	
☒	☐	ChIP-seq
☐	☒	Flow cytometry
☒	☐	MRI-based neuroimaging

Antibodies

Antibodies used	The following antibodies were used in this study: PE anti-human CD274 (#329706; 1:200; Biolegend), PE anti-mouse CD274 (#124308; 1:200; Biolegend), PE Mouse IgG2b (isotype control) (#400312; 1:200; Biolegend), Zombie Violet™ Fixable Viability Kit (#423114; 1:200; Biolegend), PerCP/Cyanine5.5 anti-mouse CD45 (#103132; 1:200; Biolegend), PE/Cyanine7 anti-mouse CD3 (#100320; 1:200; Biolegend), FITC anti-mouse CD8 (#100706; 1:200; Biolegend), APC anti-human/mouse Granzyme B (#372204; 1:200; Biolegend), PD-L1 (ab213524, 1:1000; Abcam), PD-L1 (66248-1-Ig, 1:1000; Proteintech), β-TrCP (D13F10) (#4394, 1:1000; Cell Signaling Technology), EGFR (D38B1, 1:1000; Cell Signaling Technology), Phospho-EGFR (Tyr1068, 1:1000; Cell Signaling Technology), Ubiquitin (P4D1) (#SC-8017, 1:200; Santa Cruz Biotechnology), K48 (05-1307, 1:1000; Milipore), GSK3α (#4337, 1:1000; Cell Signaling Technology), Phospho-GSK3α (Ser21) (#9631, 1:1000; Cell Signaling Technology), GSK3β (Y174) (ab32391, 1:5000; Abcam), Phospho-GSK3β (Ser9) (#P49841, 1:1000; Cell Signaling Technology), AKT (#9272, 1:1000; Cell Signaling Technology), Phospho-AKT (Ser473) (#4046, 1:2000; Cell Signaling Technology), ARIH1 (C-7) (#SC-514551, 1:200; Santa Cruz Biotechnology), ARIH1 (Goat) (#EB05812, 1:100; Everestbiotech), GST (B-14) (#SC-138, 1:200; Santa Cruz Biotechnology), Granyzme B (D6E9W) (#46890, 1:50; Cell Signaling Technology), His-tag (#66005-1-Ig, 1:1000; Proteintech), Flag-tag (0912-1, 1:2000; HuaAn Biotechnology), HA-tag (0906-1, 1:2000; HuaAn Biotechnology), β-Actin (M1210-2, 1:2000; HuaAn Biotechnology). The anti-human phospho-PD-L1 Ser279 antibody was raised against the region near Ser279 phosphorylation site of PD-L1. The secondary antibodies for western blot were used: goat anti-mouse (1:20000, #31430, Thermo Fisher Scientific, Ltd.), goat anti-rabbit (1:20000, #31460, Thermo Fisher Scientific, Ltd.).
Validation	For all the antibodies, we carried out western blot according to the method on the company's website, and detected whether the band size met the expectation with molecular weight marker, and added appropriate positive control and negative control. For EGFR, GSK3α, GSK3β, ARIH1 and so on, we added siRNA knockdown verification. Antibodies purchased from Cell Signaling

Technology were validated as per their website stating "Antibody signal is measured in model systems with known presence/absence of target signal. Includes wild-type vs. genetic knockout, targeted induction or silencing."

Eukaryotic cell lines

Policy information about cell lines

Cell line source(s)	HEK293T, U937, H1975 were obtained from ATCC. Peritoneal derived macrophages (PDMs) were obtained from male BALB/c mice.
Authentication	All cell lines were authenticated by providers (STR profiling).
Mycoplasma contamination	All used cell lines were tested negative for contamination.
Commonly misidentified lines (See ICLAC register)	None of commonly misidentified cell lines has been used.

Animals and other organisms

Policy information about studies involving animals; ARRIVE guidelines recommended for reporting animal research

Laboratory animals	Female BALB/c mice or nude mice (aged 8-10 weeks) were purchased from Shanghai SLAC Laboratory Animal Co., Ltd. (Shanghai, China). All the animal experiments were strictly conducted in accordance with the protocols approved by the Ethics Committee for Animal Studies at Zhejiang University, China. All mice were cultured in suitable temperature and humidity environment, and fed with sufficient water and food. （25 ℃， suitable humidity (typically 50%)， 12 hour dark/light cycle）
Wild animals	We don't use wild animals
Field-collected samples	no field collected samples were used in the study.
Ethics oversight	All animal studies and experimental procedures were approved by the Animal Care and Use Committee of the animal facility at Zhejiang University.

Note that full information on the approval of the study protocol must also be provided in the manuscript.

Human research participants

Policy information about studies involving human research participants

Population characteristics	EGFR-WT tumors and EGFR-mutant-driven tumors from primary lung adenocarcinoma tissues were obtained from 8 patients (4 cases each group, median age: 60 years old, range from 47 to 81).
Recruitment	All cancer patients volunteers were from Run Run Shaw Hospital of Zhejiang University.
Ethics oversight	The study was approved by the Sir Run Run Shaw Hospital of Zhejiang University School of Medicine Ethics Committee.

Note that full information on the approval of the study protocol must also be provided in the manuscript.

Flow Cytometry

Plots

Confirm that:

☒ The axis labels state the marker and fluorochrome used (e.g. CD4-FITC).

☒ The axis scales are clearly visible. Include numbers along axes only for bottom left plot of group (a 'group' is an analysis of identical markers).

☒ All plots are contour plots with outliers or pseudocolor plots.

☒ A numerical value for number of cells or percentage (with statistics) is provided.

Methodology

Sample preparation	The excised tumors were digested in collagenase/hyaluronidase (Stemcell Technologies, Vancouver, BC, Canada) and DNase (Sigma) at 37°C for 45 min to make cell suspension with a 45 µm filter (BD Bioscience). Then cells were stained with Percp-Cy5.5 conjugated-CD45, PE-Cy7 conjugated-CD8, FITC conjugated-CD3 antibodies, fixed and permeabilized with a Fix/Perm kit (Biolegend), and finally stained with APC conjugated-GzmB antibody.
Instrument	CytoFlex analyzer (Beckman Coulter)
Software	FlowJo X, CytExpert v2.3 and GraphPad Prism 7.0

Cell population abundance	moderate
Gating strategy	In our experiment, zombie was used to gate the living cells, Percp-Cy5.5 conjugated-CD45 antibody was used to gate the immune cells, FITC conjugated-CD3 antibody was used to gate the T cells, PE-Cy7 conjugated-CD8 antibody was used to circle the Tc cells, and APC conjugated-GzmB antibody was used to gate the activated Tc cells.

☒ Tick this box to confirm that a figure exemplifying the gating strategy is provided in the Supplementary Information.
